# Genotypes and different clinical variants between children and adults in progressive familial intrahepatic cholestasis: a state-of-the-art review

**DOI:** 10.1186/s13023-025-03599-2

**Published:** 2025-02-21

**Authors:** Giovanni Vitale, Marco Sciveres, Claudia Mandato, Adamo Pio d’Adamo, Angelo Di Giorgio

**Affiliations:** 1 Internal Medicine Unit for the Treatment of Severe Organ Failure, IRCCS Azienda Ospedaliero-Universitaria di Bologna, 40138 Bologna, Italy; 2 Epatologia e Clinica dei Trapianti, Ospedale Pediatrico IRCCS Bambino Gesù, Rome, Italy; 3 Dipartimento di Medicina, Chirurgia e Odontoiatria “Scuola Medica Salernitana”, Section of Pediatrics, Baronissi (Salerno), Italy; 4 Department of Medicine, Surgery and Health Sciences, University of Trieste, Trieste, Italy; 5 Institute for Maternal and Child Health—IRCCS “Burlo Garofolo”—Trieste, 34137 Trieste, Italy; 6 Pediatric Hepatology Gastroenterology and Transplantation, Hospital Papa Giovanni XIII, Bergamo, Italy; 7 Department of Medicine, Hospital Santa Maria della Misericordia, University of Udine, Udine, Italy

**Keywords:** Adult, Bile acids, Cholestasis, Intrahepatic, Drug therapy, Paediatric, Progressive familial intrahepatic cholestasis, Pruritus, Quality of life, Recurrent

## Abstract

**Introduction:**

Progressive Familial intrahepatic cholestasis (PFIC) are rare disorders of bile acid (BAs) secretion and transport with a genetic background. PFIC are paediatric manifestations, but the same variants causing PFIC can also cause cholestasis with a later paediatric onset or adult-onset cholestatic disease (AOCD). Pruritus is a symptom of cholestasis that can be so devastating that it requires a liver transplant (LT) in children; some PFIC types have been described as at risk of liver cancer development. Commonly prescribed medications for PFIC symptoms can partially relieve pruritus without changing the natural history of the disease. Recently, a therapy reducing the intestinal resorption of BAs has been approved; it is effective on both pruritus and cholestasis in PFIC, potentially being a disease-modifying intervention.

**Areas covered:**

The clinical and genetic characteristics of different PFIC and AOCD are summarized to provide a common background for geneticists and paediatric and adult hepatologists in diagnosis and management.

**Expert opinion:**

Collaboration between paediatric and adult hepatologists and geneticists will become crucial for cholestatic disease research and patient treatment. Therefore, adult hepatologists will need to learn more about FIC. This might enable the implementation of individualized surveillance in FIC patients and the evaluation of patient family histories.

## Article highlights

- Progressive familial intrahepatic cholestasis (PFIC) are rare, known paediatric-onset disorders of BA secretion and transport whose diagnosis is based on clinical symptoms to be confirmed with genetic tests that often reveal homozygosity or compound heterozygosity.
- Several adult-onset cholestasis share mutations in PFIC genes, usually as single heterozygosity, albeit presenting with different phenotypes compared to children-onset PFIC.
- Although adult-onset PFIC phenotypes generally progress less rapidly and are less severe, there is a significant risk of gallstones, intrahepatic cholestasis of pregnancy, drug-induced liver injury, fibrosis, cirrhosis, and liver malignancy. After the clinical observation of phenotypes attributable to PFIC, current research techniques enable the isolation of novel variants related to known genes or variants in other genes that determine known or new cholestatic intrahepatic phenotypes.
- In children with PFIC, new treatments based on BA transporter inhibitors allow for controlling the pruritus symptom and cholestasis, making native liver survival more likely.
- BAs transporter inhibitors are indicated in paediatric patients in PFIC phenotypes.
- The importance of early and accurate diagnosis is becoming crucial. There is a continuum in patient management, with paediatric to adult hepatologists collaborating with geneticists.
- Educational programs to ensure the recognition of PFIC genes in adult-onset cholestasis will be mandatory to transform the new knowledge into a broad improvement for the patients and their families.

## Introduction

Familial Intrahepatic Cholestasis (FIC) represents a heterogeneous group of genetic disorders affecting bile acid (BA) secretion and transport [1]. Progressive Familial Intrahepatic Cholestasis (PFIC) refers to paediatric-onset manifestations, but the same genetic variants can also cause adult-onset cholestatic disease (AOCD) [1]. There are several different PFIC types related to different gene mutations and extreme phenotypic variability [2]. Estimated prevalence ranges from 1 per 50,000 to 1 per 100,000 births [3]. However, the exact prevalence of newer variants of PFIC has yet to be discovered due to the limited number of studies, mostly case reports or small case series [2]. Patients with PFIC may present with a wide variability of signs and symptoms, including cholestasis, jaundice, and pruritus; symptoms occur during the neonatal period, infancy, or early childhood, as well as during adolescence and adulthood. Biochemical features of PFIC include high serum bile acids (BAs) and low γ-glutamyl transferase (γGT) levels in the majority of cases [4–8]. PFIC typically causes progressive fibrogenic liver disease, leading to portal hypertension, cirrhosis, and finally to end-stage disease with liver failure requiring liver transplantation (LT) [9].

Pruritus is one of the main symptoms of cholestasis in many PFIC patients [2]; it is often very severe and may unfavourably affect the sleep, social life, learning, and quality of life (QoL) of patients and their carers [10]. In severe cases, pruritus can necessitate liver transplantation [2].

These diseases are linked to gene variants encoding proteins that maintain biliary epithelium integrity and determine hepatocyte cell membrane composition [2]. Additionally, certain genes encode proteins that either form membrane channels or are cellular components, ensuring the proper localization of these channels [9]. In some cases, [11] the relationship between the gene and the disease needs to be better understood. Numerous genes linked to the known 13 different types of PFIC have been discovered so far [12].

The treatment of PFIC has long been based on the use of drugs, such as ursodeoxycholic acid (UDCA), rifampicin, and cholestyramine, or surgical biliary diversion (SBD), aimed at reducing symptoms, especially pruritus [13]. However, none of these drugs are approved for the treatment of PFIC by the European Medicines Agency (EMA) (except for UDCA, which is approved in France for the treatment of PFIC3) [14] and their efficacy is debated [1], being often unable to avoid disease progression and listing for LT [6, 8]. Less than half of PFIC1 patients were reported to reach adulthood with the native liver, while about 70% of patients underwent SBD; a retrospective study on PFIC2 patients reported that 18 out of 48 patients had SBD while 22 out of 48 had LT [15]. Odevixibat, a small molecule that inhibits ileal resorption of BAs belonging to the class of ileal BAs transporter (IBAT) inhibitors, was the first drug approved for the treatment of PFIC patients ≥ 6 months old due to its efficacy on both pruritus and reduction of serum BAs as well as its good tolerability in 2021 [16]. Thanks to the increased use of genetic testing, some AOCDs have been found to be associated with PFIC gene mutations, with symptoms of varying severity and forms of intermittent cholestasis traditionally considered to be benign or not progressive [1]; these are:

- LPAC (Low-Phospholipid Associated Cholelithiasis) [17]
- RIC (Recurrent Intrahepatic Cholestasis) [18]
- DIC (drug-induced cholestasis) [19, 20]
- ACC (Adult Cryptogenetic Cholestasis [21, 22]
- ICP (Intrahepatic Cholestasis of Pregnancy [23]
- Cofactor of progression in other liver diseases [24]
- HBC (Hepatobiliary cancer) [9]

While in many cases the symptoms of these diseases can be controlled for a long time, in other cases the patient can undergo rapid progression, fibrosis, or cancer [9].

Concerning the risks of developing hepatobiliary cancers in FIC-related diseases, they are due to an overexpression of pro-inflammatory cytokines, resistance to apoptosis, and, ultimately, cell hyperproliferation [25].

The genes involved in vivo and in vitro studies in the risk of development of hepatocellular carcinoma (HCC) and cholangiocarcinoma (CCA) are ABCB11, ABCB4, TJP2, FXR, MYO5B, SLC51B, SLC25A13, NOTCH2, JAG1, TGR5 and HNF1B both in paediatric and non-paediatric populations [9]. ABCB11 (PFIC2) variants present an enhanced risk of developing liver cancers, as shown in the NAPPED study [15].

AOCDs are frequently associated with the same genes that determine PFIC, but while in PFIC these genes behave as recessive, in adult cholestasis, they can behave as autosomal dominant, being often in the heterozygous state [22].

This review focuses on the pleiomorphic clinical presentations and the genetic substrate of FIC and proposes a possible diagnostic and therapeutic work-up for this group of diseases, considering the shared factors and therapeutic novelties that may affect them.

## Characteristics of PFIC and PFIC-related diseases in paediatric populations

PFIC causes progressive intrahepatic cholestasis in newborns, infants, and children. Byler’s disease, the first disease attributable to a progressive form of intrahepatic cholestasis in childhood, was described in the mid-1960s in 11 Amish children from 6 families [26]. Since then, 12 forms of PFIC have been identified; the three most known forms (PFIC1 [27], 2 [28], and 3 [29]) have been identified through immunochemistry and Sanger sequencing [30]. Sanger sequencing is nowadays utilized as a method to confirm mutations in specific genes when clinical evidence strongly suggests a particular subtype [31, 32]. After the introduction of the New Generation Sequencing (NGS) technology [30], which relies on platforms for high-throughput, massively parallel sequencing that can analyse many genes at once, it was possible to identify the forms of PFIC, from PFIC1 to PFIC13 [4, 8, 11, 33–37].

NGS is being used in clinical settings with various approaches that vary in depth, cost, and timing. To streamline gene analysis and lower sequencing costs, NGS can be employed to examine only specific genes of interest, using targeted gene panels available on the market or prepared in the lab; on the other side, the Whole Exome Sequencing (WES) and Whole Genome Sequencing (WGS) methods are the most comprehensive. WES analyses encode regions and exon–intron junctions of known genes, while WGS, the most complex and expensive analysis, looks for coding and non-coding regions and is used in research or complex cases [38]. Typically, WES is pursued when examining genes associated with PFIC, which yields a negative result. In such instances, all genes are analysed with the hope of identifying a new cholestasis gene [39]. If a new gene is identified, subsequent functional studies would be necessary to confirm the pathogenicity of its mutations [40].

The following paragraphs summarize the characteristics of the 13 forms of paediatric PFIC described so far.

## Different phenotypes: PFIC in paediatric age

### PFIC1

PFIC1 is a condition that usually shows symptoms within the first three months of life. It is caused by variations in the ATP8B1 gene, which encodes transmembrane lipid transporter proteins located in the membrane. These proteins, known as flippases or FIC1, are involved in maintaining an asymmetric distribution of phospholipids across the canalicular membrane of hepatocytes, thereby protecting the canalicular membrane from hydrophobic BAs and maintaining its integrity. γGT and α-fetoprotein (αFP) levels are usually normal, BAs are elevated, and alanine aminotransferase (ALT) levels are less than five times the upper limit of normal; pruritus is severe, while jaundice is moderate. Liver ultrasonography is usually normal but can reveal a massive gallbladder. Liver histology demonstrates canalicular cholestasis and the absence of genuine ductular growth, with only periportal biliary metaplasia of hepatocytes. Cholangiography, when performed, reveals a normal biliary tree. Biliary lipid analysis can highlight a modestly reduced biliary salt content [1, 2, 41, 42].

## PFIC2

PFIC2 is caused by variants in the ABCB11 gene, which encodes the bile salt export pump (BSEP), the primary transporter of BAs from hepatocytes to the canalicular lumen. Clinical indications of cholestasis (discoloured faeces, dark urine) frequently occur in the first few months of infancy (with a tendency to appear earlier than PFIC1), with recurring or persistent jaundice, low γGT, elevated BAs and αFP, hepatomegaly, and severe pruritus. Early onset of liver failure and/or development of HCC during childhood may worsen the course of PFIC2. Typically, patients develop fibrosis and end-stage liver disease before reaching adulthood; advanced fibrosis and cirrhosis are conditions predisposing to hepatobiliary cancers (HBCs) [14]. Early therapy with UDCA or SBD may reduce morbidity and mortality associated with end-stage liver disease. Furthermore, individuals may develop biliary stones, DIC, and/or ICP later in the disease course [1, 2, 42, 43].

### PFIC3

PFIC3 is caused by variants in the ABCB4 gene, which encodes multidrug-resistance protein 3 (MDR3/ABCB4); this protein transports phospholipids into the canalicular lumen to neutralize bile salts and prevents injury to biliary epithelia and bile canaliculi. Patients have high γGT levels, normal cholesterol levels, and normal or mildly elevated BA concentrations. Other symptoms are severe jaundice, diarrhoea, fever, pruritus, and hepatosplenomegaly. Ultrasonography of the liver is usually normal; however, it can indicate a large gallbladder and sometimes biliary stones. Histology of the liver reveals portal fibrosis and genuine ductular growth, as well as a mixed inflammatory infiltration and, eventually, evidence of biliary cirrhosis. Cholangiography reveals a normal biliary tree, allowing sclerosing cholangitis to be ruled out. Biliary lipid analysis reveals lower biliary phospholipid levels. Patients with PFIC3 might not show symptoms until 2–3 years old, unlike those with PFIC1 and PFIC2 [1, 2, 42, 44].

### PFIC4

PFIC4, a paediatric cholestasis with low γGT and elevated BAs, is caused by variants in the TJP2 gene, which encodes the protein known as tight junction protein 2 or zona occludens-2 (ZO-2), involved in maintaining cell-to-cell adhesion. The age of onset ranges from the first days of life to a few months, with jaundice and hepatomegaly [33]. The underlying pathogenetic mechanism leading to cholestasis in PFIC4 has yet to be completely understood. In TJP2 variants, the claudine CLDN1 fails to position itself at the bile duct membrane, causing the reflux of toxic BAs into hepatocytes, hepatocyte damage, and cholestasis [45, 46]. The high response rate to treatment with IBAT (odevixibat) in these patients suggests that in PFIC4, despite tight junction dysfunction, there is a regular passage of BAs from the hepatocytes to the biliary tree and the bowel, allowing odevixibat to interrupt the enterohepatic circulation and decrease the recirculating BA pool [1, 42, 47].

### PFIC5

PFIC5, paediatric cholestasis with jaundice, low γGT, and elevated BAs, whose onset age ranges from a few days to a few weeks [48], is related to a deficiency of the BA receptor known as farnesoid X receptor (FXR) due to loss of function variant in the NR1H4 gene. In the liver, the FXR is a BA-sensing receptor involved in the expression of BSEP and is also expressed in the small intestine; the NR1H4 variants cause loss of BSEP expression, leading to the accumulation of toxic bile and hepatocellular damage, with rapidly progressive intralobular cholestasis in the neonatal period [1, 42, 48, 49].

### PFIC6

A homozygous mutation in the SLC51A gene causes PFIC6. Solute carrier family 51 alpha subunit (SLC51A) encodes the OSTα-OSTβ complex, involving intestinal BAs reabsorption in the enterohepatic circulation. Gao [5] reported a Pakistani child with jaundice and chronic malabsorptive diarrhoea. Laboratory tests highlighted elevated transaminases, γGT, and alkaline phosphatase (ALP), while liver histology showed portal and periportal fibrosis and hepatocytes with foci of cholestasis and normal BA levels [5, 42]. Two Palestinian brothers were reported to have jaundice and diarrhoea that started soon after birth [6].

### PFIC7

PFIC7 is caused by variants in the USP53 gene, which encodes for Inactive ubiquitin carboxyl-terminal-hydrolase-53, involved in the degradation of proteins; the phenotype related to USP53 mutation is probably related to a defective tight junction complex. Age of onset ranges from infancy to adolescence; cholestasis is generally mild and intermittent, with pruritus, normal γGT, elevated BAs, and transaminases, but liver fibrosis is often present [42, 50, 51].

### PFIC8

PFIC8 is caused by variants in the KIF12 gene, encoding for a microtubule motor protein. KIF12-associated impaired functional cell polarity may be the underlying cause. The associated phenotype includes fibrosis, cholestasis, jaundice, bile duct proliferation, and elevated γGT and ALP; onset is in neonatal age, and symptoms range from neonatal cholestasis with complete clinical remission or absence of clinical symptoms with the diagnosis made incidentally to a progressive disorder requiring LT [7, 42, 51].

### PFIC9

PFIC9 is caused by a variant in the ZFYVE19 gene that encodes for the Zinc Finger FYVE-Type Containing 19 protein; the variant results in a ciliopathy with elevated γGT. The phenotype is chronic cholestasis [8] with onset in infancy or early childhood. The reported affected individuals have neonatal cholestasis with severe pruritus and hepatosplenomegaly; they may have portal hypertension or upper gastrointestinal bleeding. The liver biopsy shows fibrosis, cirrhosis, bile duct proliferation, and abnormal bile duct morphology. The disorder is thought to result from ciliary defects in cholangiocytes. ZFYVE19 is expressed ubiquitously. However, the clinical phenotype is prevalently characterized by hepatopathy [8, 52]. Odevixibat treatment was effective in reducing pruritus and BA levels in a PFIC9 patient [42, 53].

### PFIC10

PFIC10 is caused by a variant in the MYO5B gene, encoding for myosin-Vb protein, a carrier protein essential for plasma membrane recycling and epithelial cell polarization [35]. The onset of symptoms is in the first months or years of life. Features include jaundice, pruritis, diarrhoea, and hepatomegaly associated with increased serum bilirubin and BAs. Liver transaminases may be variably increased while γGT is normal. This phenotype usually does not include microvillous inclusion disease (MVID), which the MYO5B mutation is known to cause [33], but is generally limited to cholestasis [35, 42, 54].

### PFIC11

PFIC11 is driven by a mutation in the SEMA7A gene that encodes for Semaphorin-7A; this is likely a gain-of-function variant that reduces BSEP and Mrp2 expression in hepatocytes [11]. Semaphorin-7A is a membrane-bound protein that involves axon growth and other biological processes. SEMA7A variants were found to be associated with familial cholestasis, jaundice, normal γGT, and elevated levels of serum transaminases and BAs in a female infant born of unrelated Chinese Han parents [11]. SEMA7A variants in animal models reduce the levels of canalicular membrane BAs transporters, like BSEP, in hepatocytes [11, 42].

### PFIC12

PFIC12 is caused by a variant in the VPS33B gene, encoding the vacuolar sorting-associated protein 33B; the mutated gene can cause isolated cholestasis with low γGT, neonatal-onset jaundice, and conjugated hyperbilirubinemia, associated with intense pruritus and hepatosplenomegaly [36, 42].

### PFIC13

Recently, a thirteenth form of PFIC was identified by means of a survey involving 279 families and 299 patients with intrahepatic cholestasis; in 4 families, the PSKH1 gene was identified; 3 families were related. PFIC13 has a phenotype of hepatorenal ciliopathy; the patient’s fibroblasts showed abnormally long cilia with abnormal transport [37].

The malfunction of these genes causes impaired production and excretion of bile, resulting in cholestatic liver disease. Biliary substances cannot be eliminated from the liver and, thus, re-enter the general blood circulation. This results in the deposition of bilirubin pigments in the tissues and ultimately causes jaundice. Pruritus, the most unbearable symptom in cholestasis, is probably induced by the stimulation of nonmyelinated subepidermal free nerve ends because of increased levels of serum BAs [13].

The characteristics of paediatric PFIC are summarized in Table 1.

**Table 1:** Characteristics of pediatric PFIC

	Locus/gene/protein	Clinic	BAs	γGT	Age of onset	Histology
PFIC1	ATP8B1 FIC1	Severe pruritus, moderate jaundice, Pancreatic disease Rickets, Pneumonia Abnormal sweat test Short stature Watery diarrhoea Sensorineural deafness Extrahepatic cystic fibrosis Alzheimer’s disease Hypothyroidism	High	Normal	Within the first 3 months of life	Canalicular cholestasis. Absence of genuine ductular growth, periportal biliary metaplasia of hepatocytes
PFIC2	ABCB11 BSEP	Jaundice, hepatomegaly, severe pruritus Skin thickening Light dysmorphic features Slow growth Early onset of liver failure Fibrosis and end-stage liver disease before adulthood; advanced fibrosis and cirrhosis predispose to HBCs	High	Normal	First months of life	No hepatobiliary structural abnormality. Amorphous or finely filamentous bile, giant cell hepatitis
PFIC3	ABCB4 MDR3	Severe jaundice, diarrhoea, fever, pruritus, hepatosplenomegaly Mental impairment Growth retardation Reduced bone density possible predisposition to HCC in early childhood	Normal or mild elevation	High	2–3 years	Portal fibrosis and genuine ductular growth, mixed inflammatory infiltration
PFIC4	TJP2ZO-2	Jaundice, hepatomegaly, Subdural hematomas Chronic respiratory disease possible predisposition to HCC in early childhood	High	Normal	First days- a few months of life	Lack of TJP2 protein expression, damage to tight junctions
PFIC5	NR1H4 FXR	Rapidly progressive intralobular cholestasis in the neonatal period Vitamin K-independent coagulopathy	High	Normal	From a few days to a few weeks	Intralobular cholestasis, diffuse giant cell transformation, ballooning hepatocytes, and ductular reaction
PFIC6	SLC51A OSTα-OSTβ	Elevated transaminases, cholestasis, Chronic malabsorptive diarrhoea Easy bruising Episodes of prolonged bleeding	Normal	High	A few days after birth	Portal and periportal fibrosis
PFIC7	USP53 Inactive ubiquitin carboxyl-terminal-hydrolase-53	Mild and intermittent cholestasis, liver fibrosis, pruritus, hearing loss, jaundice, elevated AST, and ALT elevated ALP, hypocalcemia	High	Normal	From infancy to adolescence	Hepatocellular and canalicular cholestasis with fibrotic changes
PFIC8	KIF12 Microtubule motor protein	Neonatal cholestasis, jaundice; persistently elevated γGT and ALP	High	High	Neonatal age	Fibrosis, bile duct proliferation, fibrosis, cirrhosis
PFIC9	ZFYVE19 Zinc Finger FYVE-Type Containing 19	Hepatosplenomegaly, portal hypertension, upper gastrointestinal bleeding, diarrhea	High	High	Infancy or early childhood	Fibrosis, cirrhosis, bile duct proliferation, and abnormal bile duct morphology
PFIC10	MYO5B Myosin-Vb	Jaundice, pruritis, hepatomegaly Microvillus inclusion disease (MVID) leads to intractable diarrhoea Language development delay Pyramidal syndrome	High	Normal	First months or years of life	Hepatocellular and canalicular cholestasis with giant cell changes
PFIC11	SEMA7A Semaphorin-7A	Jaundice, no itching	High	Normal	Infancy	In a mice model, the same mutation resulted in cholestatic liver disease associated with decreased levels of BA transporters
PFIC12	VPS33B vacuolar sorting-associated protein 33B	Jaundice, intense pruritus. Hepatosplenomegaly, mildly prolonged aPTT Arthrogryposis Renal Dysfunction-Cholestasis (ARC) syndrome	High	Normal	First weeks of life	Cholestasis with giant cell formation

PFIC, Progressive familial intrahepatic cholestasis; HCC, Hepatocelllar carcinoma; γGT, Gamma-glutamyl transferase; ALP, Alkaline phosphatase; aPTT, activated partial prothrombin time

## Extrahepatic manifestations of paediatric PFIC

**Fig. 1:**
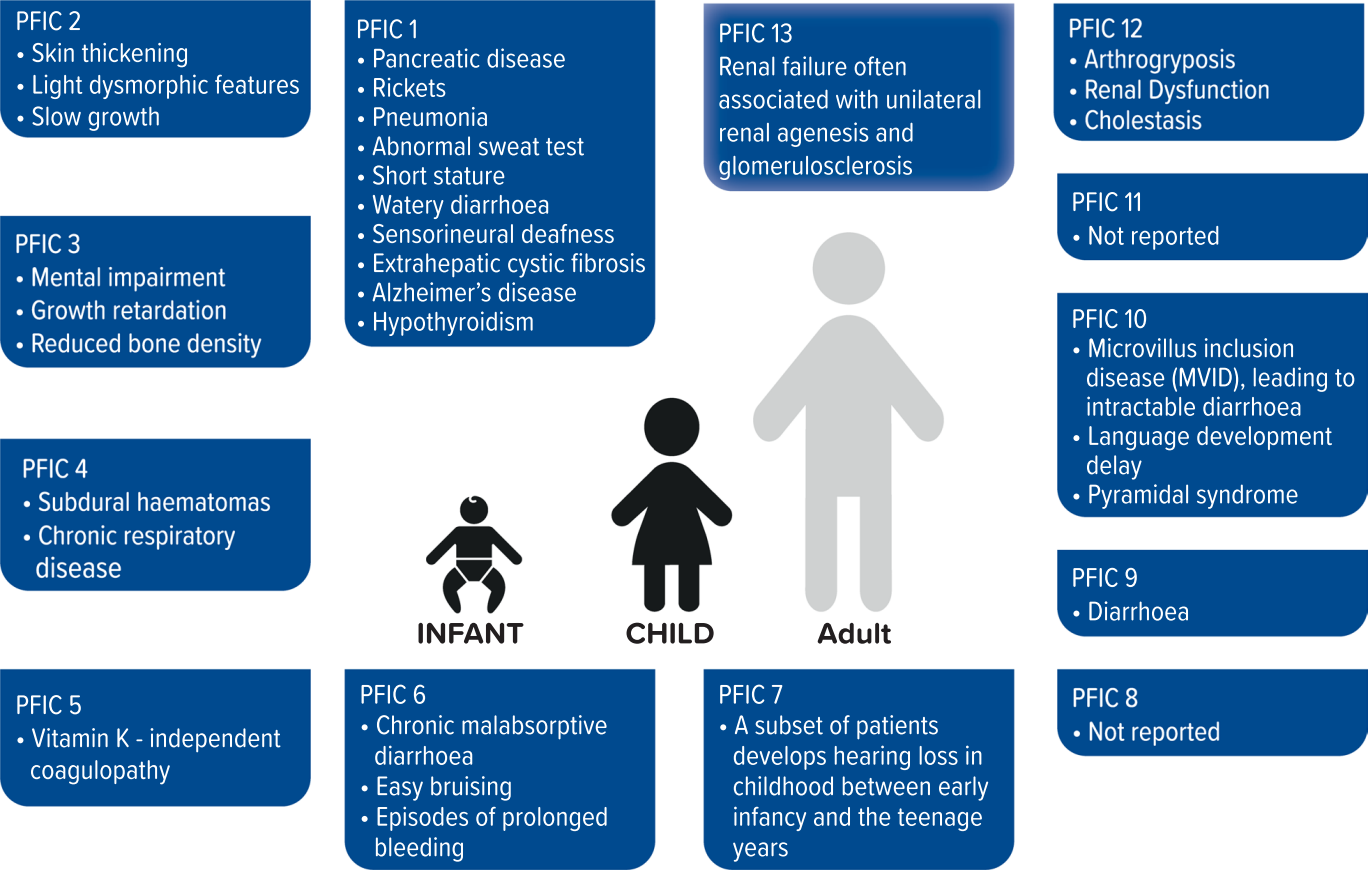
Extrahepatic manifestations of progressive familial intrahepatic cholestasis syndromes in infants and children

Extrahepatic symptoms are often observed in paediatric patients with PFIC subtypes if the affected gene is highly expressed in tissues other than the liver [55] (Fig. 1).

The FIC1 gene (ATP8B1) encodes an ATPase, which is expressed not only in the liver and small intestine but also in pancreatic acinar cells, gastric pit epithelial cells, and cholangiocytes as well as in the inner ear; extrahepatic manifestations, such as diarrhoea, pancreatic disease, rickets, pneumonia, abnormal sweat tests, hearing impairment, and poor growth have been described in PFIC1 patients [2]. In a study comparing 42 patients with FIC1 disease to 60 patients with BSEP disorder, PFIC1 patients suffered from symptoms like diarrhoea, allograft steatosis, and pancreatic disease and were less likely than BSEP disease patients to catch up with weight gain following LT [55]; the outcome following LT remained unsatisfactory in many FIC1 disease patients.

Unlike ATPB8B1, the gene responsible for PFIC2 (ABCB11) is expressed only in the canalicular membrane of the hepatocyte; consequently, extrahepatic manifestations related to this mutation (diarrhoea, fever) are scarce compared to those related to ATP8B1 [55]. They may include skin thickening, light dysmorphic features, slow growth [42].

154 different FIC1, BSEP, and MDR3 variants were found in a German cohort of 427 patients, 25 of which were novel. These common variants may be involved in a cholestatic phenotype, as BSEP and MDR3 polymorphisms were significantly overrepresented in patients without disease-causing mutations in the respective genes [56].

Variants of the ABCB4 gene, encoding for MDR3, are related to a broad spectrum of liver diseases, but also to mental impairment, growth retardation and reduced bone density, and, notably, to an increased probability of developing CCA [42, 55].

PFIC 4 patients are very rare; subdural hematomas and chronic respiratory diseases were reported in two patients, the latter per the increased expression of TJP2 in the lung [45, 55].

NR1H4 mRNA is expressed across a wide range of tissues; thus, it is not surprising that children with PFIC5 suffer from a wide range of extrahepatic manifestations. Four patients were reported to have severe vitamin K-independent coagulopathy early in the course of their disease. This is consistent with the role of FXR in coagulation [33, 42].

PFIC6 gene (SLC51A) malfunctioning in PFIC patients seems to be related to chronic malabsorptive diarrhoea, coagulopathy, fat malabsorption, severe fat-soluble vitamin deficiency, rickets, and mild liver involvement [5].

USP53, the gene responsible for PFIC7, was found to be related to hearing loss and hypocalcemia [51].

Regarding PFIC9, the patients seem to have no extrahepatic manifestations apart from diarrhoea [8, 42, 52].

PFIC10, a biallelic mutation in the MYO5B gene, presents with variable severity of symptoms and sometimes is related to MVID [57, 58], leading to intractable diarrhoea; language development delay and pyramidal syndrome were also reported [42].

In some PFIC12 (caused by a variant in the VPS33B gene) patients, arthrogryposis, Renal Dysfunction-Cholestasis (ARC) syndrome, and mildly prolonged aPTT have been observed [36].

## Intrahepatic cholestasis in the young and adult population

While PFIC are characteristic of the paediatric and adolescent population, several cholestatic diseases also affect the adult population, and often the same genes that determine PFIC in children are involved in different ways. PFIC are inherited in an autosomal recessive manner, indicating that patients carry mutations in both alleles of the disease gene. Contrastingly, in adults, mutations are often identified in just one of the two alleles, hinting at a haploinsufficiency mechanism. Such mutations can lead to lifelong controlled conditions or disorders that progress abnormally and swiftly [51, 52]. Familial intrahepatic cholestatic diseases could, therefore, be considered as a spectrum in which the paediatric PFIC is the extreme characterized by the most severe manifestations. At the same time, variants in these genes in the heterozygous state can cause numerous cholestatic pathologies in adults, with different severity and tendencies to progression.

Among the 356 adult patients of a study on variants in cholestasis-related genes in adults, 101 were identified as carriers of variants of the genes ATP8B1, ABCB11, and ABCB4, often in heterozygosity. RIC and ICP were identified during the investigation of the family history of some patients. More than 70% of patients with variants of ABCB11 and ABCB4 had fibrosis [22]. In another study on 48 patients with ACC [21], pathogenic/likely pathogenic mutations and polymorphisms of the ATP8B1, ABCB11, ABCB4, and TJP2 genes were identified in 21%. Patients with pathogenic/likely pathogenic mutations had more frequently a history of neonatal jaundice, with increased BAs and increased presence of fibrosis. Multiple mutations were present in more aggressive phenotypes with a synergistic effect.

Given the significant variability of the manifestations, an elevated degree of suspicion and clues in the family history are needed to detect these pathologies in the adult population.

### Low phospholipid associated cholelithiasis

LPAC is the most frequent cause of gallbladder stones in the young population [17]; biliary colic and acute cholangitis are symptoms. Diagnostic criteria for LPAC are the onset of symptoms before the age of 40 years, the recurrence of symptoms after cholecystectomy, and intrahepatic microlithiasis; an LPAC diagnosis should be considered when at least two of them are met [59]. ABCB4, responsible for PFIC3, is the gene mutation most frequently associated with LPAC, followed by ABCC2. The genetic investigation does not reveal homozygosity or compound heterozygosity, but pathogenic variants in a single copy and, sometimes, variants of uncertain significance (VUS) [17]. A retrospective study shows that, in 233 patients with juvenile lithiasis, those who have mutated ABCB4 have a greater number of abnormalities in magnetic resonance (MR), have more frequent calculosis, and greater susceptibility to advanced liver disease, CCA and secondary sclerosing cholangitis [1, 17, 42].

### Recurrent intrahepatic cholestasis

Recurrent intrahepatic cholestasis (RIC), historically also known as BRIC (benign recurrent intrahepatic cholestasis), is a disorder characterized by high levels of bilirubin and ALP, a minimum of two episodes of jaundice, and normal or nearly normal values for γGT. Age at first presentation can range from 1 to 59 years. Patients may experience attacks that last from weeks to months, followed by asymptomatic intervals that can last months or years. The triggers that can cause these episodes include pregnancy, drugs, and infections [60]. Associated variants are on ATP8B1 (responsible for PFIC1 and (B)RIC1) and ABCB11 (determining PFIC2 and (B)RIC2) genes [9], but cases linked to MYO5B variants have also been recently described [60]. Despite RIC is classified as an autosomal recessive disorder, five of the seven Japanese patients with RIC in a retrospective study were compound heterozygous, and two were simple heterozygous with a single variant of the ATP8B1 or ABCB11 genes; some variants may result in BRIC forms with dominant inheritance [61]. The episodes usually resolve spontaneously and do not lead to progressive liver injury [18]. RIC is usually benign but might be associated with an increased risk of fibrosis [11]; some patients may develop the disease at a later stage, and the clinical impact may be less severe compared to Progressive Familial Intrahepatic Cholestasis (PFIC) [18]. For this reason, we prefer to define this disease as Recurrent Intrahepatic Cholestasis (RIC) rather than a form of benign cholestasis (BRIC) in this review, given the possible risk of progression over the years. Moreover, the phenotype of the disease may evolve from episodic to more severe chronic cholestasis [62].

Mutations on the ABCB4 gene have been associated with several liver diseases (LPAC, ICP, DIC, transient neonatal cholestasis) and only anecdotally with episodic FIC in later age [1, 56, 62, 63].

### Drug-induced cholestasis

More than 40% of adult over-50 cases of hepatitis and more than 50% of cases of acute fulminant hepatic failure have been attributed to idiosyncratic drug-induced liver injury (iDILI); DIC represents about 30% of iDILI [1, 64]. DIC is more common among the elderly (> 60 years old patients) and is associated with high mortality (up to 10%); clinical presentation is highly variable, including bland cholestasis, cholestatic hepatitis, secondary sclerosing cholangitis, and vanishing bile duct syndrome (VBDS) [64]. Proteins and genes involved in DIC include BSEP (ABCB11) and MDR3 (ABCB4) [19], which in homozygous form are responsible for paediatric PFIC2 and PFIC3; up to 50% of DIC patients have ABCB4 variants [17, 19, 20, 65]. Common ABCB11 variant p.Val444Ala, able to reduce BSEP, was observed frequently in patients with DIC [64]. Moreover, the multidrug resistance protein-1 (MDR1, ABCB1), which transports organic cations, and the multidrug resistance protein-2 (MRP2, ABCC2), which regulates the independent flow of bile salts by excreting glutathione are involved [20].

Genetic predispositions, older age, elevated dosages, and drug characteristics—such as high lipophily—may be risk factors for DIC [64]. Although several other medications have been linked to DIC, antibiotics remain the leading cause of this condition [60], along with underlying liver disease: for instance, in patients with a history of ICP, there appears to be a greater predisposition toward a cholestatic liver injury from oral contraceptives or postmenopausal hormone replacement, while rifampicin seems to be associated with an increased risk for hepatotoxicity in patients with primary biliary cholangitis (PBC) [64].

**Fig. 2:**
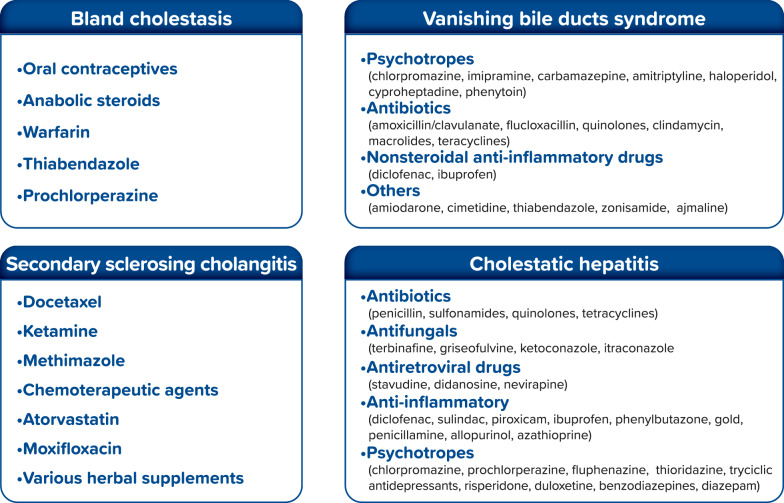
Drugs potentially involved in DIC

In most cases, when the trigger medication is discontinued, abnormal liver tests reverse to normal; VBDS has a variable clinical course, from full liver recovery and reversibility to prolonged bile duct loss [64]. Drugs involved in DIC, and their possible effects are summarized in Fig. 2.

### ICP

ICP is a pregnancy-specific liver disease that may lead to adverse fetal outcomes, including preterm delivery, meconium staining of the amniotic fluid, and stillbirth. ICP affects 0.1–2% of pregnant women in the late second and early third trimester of pregnancy; it typically affects individuals under 40 years old [42]. It is diagnosed in women with gestational pruritus and increased BAs [66]. In women with ICP, BAs levels above 40 μmol/L are associated with neonatal mortality, preterm birth, and foetal stress; heterozygous mutations in ATP8B1, ABCB11, ABCB4, TJP2, NR1H4 in ICP have been reported [9, 23]. A meta-analysis suggests that stillbirth risk increases when BA concentrations are 100 µmol/L or more [23]. The mainstay of therapeutic management consists in the reduction of maternal symptoms and possible complications to the foetus. UDCA is the most used treatment for ICP and can decrease foetal BAs levels and improve pruritus. Still, a systematic review and individual participant data meta-analysis showed no significant effect on the prevalence of stillbirth [67]. Also, rifampicin, if added to UDCA, can help improve pruritus and BAs [66]; other medications such as cholestyramine and S-adenosyl-L-methionine are treatment options [64]; effective alternative treatment is currently lacking [1, 67]. Patients with severe ICP (BAs > 100 µmol/L), recurrent ICP and/or early onset ICP should be offered genetic testing, according to the EASL guidelines on genetic cholestasis [68].

### ACC

Cryptogenic cholestasis (ACC), defined as γGT and/or ALP persistently > 1.5-fold the upper normal values in at least two tests or as a history of pruritus combined with elevated BAs (10 μmol/l) for more than 6 months, occurs in patients of different ages; after excluding other causes of the disease, it is feasible to explore the existence of mutations linked to PFIC in adults with adult-onset cryptogenic cholestasis (ACC) [21].

Cholestatic patients, 18 years or older, who had undergone genetic sequencing for cholestasis over 5 years were identified in a retrospective study [22]. 356 adult patients were examined for ATP8B1, ABCB11, and ABCB4 variants. 101 patients (28.4%) had at least one genetic variant, and 9 patients with variants in more than one gene were identified. The median age at presentation was 36.2 years.

ABCB4 variants were associated with ICP (75%) and chronic liver disease (71.7%), with more severe genotypes correlating with an earlier onset.

ABCB11 variants presented as acute/episodic cholestasis (40%) or ICP (82.4%). ATP8B1 variants were linked to chronic liver disease (75%); however, these variants, which had a low predicted pathogenicity, were common in patients with different underlying liver diseases. The patient’s family history frequently included RIC and ICP episodes.

A study on Italian outpatients [21] who had cryptogenic cholestasis for more than 6 months, aged 6 years or more, evaluated 48 patients; 21% of them had polymorphisms and pathogenic/likely pathogenic mutations in the genes ATP8B1, ABCB11, ABCB4, and TJP2. The mean age at the time of the genetic test was 42 years. Individuals with pathogenic or probably pathogenic mutations had higher levels of BAs, fibrosis, and a history of neonatal jaundice; multiple mutations together produce more aggressive phenotypes [21].

### Cofactor of progression in other liver diseases

The variants responsible for the most common and known forms of PFIC, particularly the variants of ABCB4, have been linked to various heterogeneous cholestatic diseases [24]; these variants seem to be inherited through recessive inheritance in the case of PFIC, while less severe diseases like ICP or LPAC are likely to be inherited as autosomal dominant variations.

The relationship between a pathogenic variant of ABCB4 and the progression towards fibrosis and cirrhosis of diseases such as PSC (primary sclerosing cholangitis) and PBC has been explored. Two cohorts of Polish patients, for a total of 867 (456 with PBC and 411 with PSC) were evaluated in a retrospective study; among PBC patients, carriers of the risk variant c.711A > T (widespread in the general population, both as heterozygous and as homozygous) presented more frequently with cirrhosis; during the follow-up, a total of 22 patients in PBC group developed cirrhosis, with a higher risk among carriers of this variant, in agreement with the different clinical presentation of patients with PBC and PSC [24].

A review identified phenotypes of ABCB4 deficit in addition to PFIC3, DIC, and ICP: chronic cholangiopathy, adult biliary fibrosis/cirrhosis, some cases of transient neonatal cholestasis, and parenteral nutrition-associated liver disease [69].

**Fig. 3:**
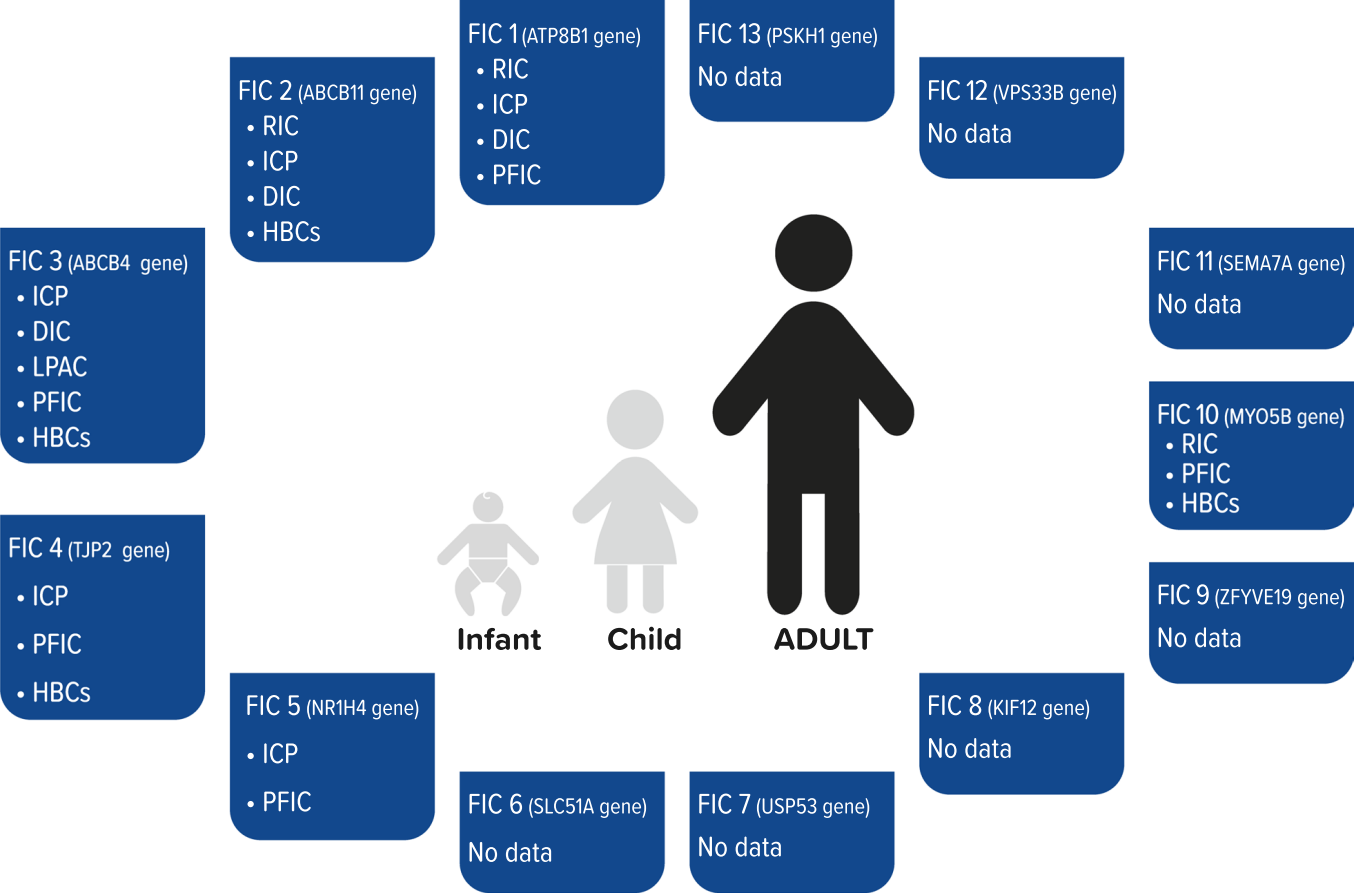
Hepatic manifestations of familial intrahepatic cholestasis syndromes in adult patients. DIC, drug-induced cholestasis; ICP, intrahepatic cholestasis of pregnancy; HBCs, hepatobiliary cancers; LPAC, low-phospholipids associated cholelithiasis; PFIC, progressive familial intrahepatic cholestasis

Genes and proteins involved in adult cholestasis and their clinics and laboratory results are summarized in Table 2; hepatic manifestations of FIC genes in adult patients are summarized in Fig. 3.

**Table 2:** Genes and proteins involved in adult cholestasis

	Locus/gene/protein	Clinics	BAs	γgt	ALP	AST/ALT	Histology
Recurrent Intrahepatic cholestasis (RIC)	ATP8B1/FIC1 ABCB11/BSEP MyosinVB/MYO5B	Intermittent severe cholestasis (intervals weeks to years); hearing loss, pancreatitis, diarrhoea	High during attack	Low or normal	High	Normal or mild elevation	Centrilobular cholestasis, no alteration of liver structure, no BSEP tissue expression
Intrahepatic cholestasis of pregnancy (ICP)	ATP8B1/FIC1 ABCB11/BSEP ABCB4/MDR3 TJP2/ZO-2	Transient cholestasis + itching during pregnancy; post-natal resolution; potentially serious fetal complications	High during pregnancy	Normal or mild elevation	Normal or mild elevation	Normal or mild elevation	Not performed
Drug-induced cholestasis (DIC)	ABCB11/BSEP ABCB4/MDR3	Chronic liver injury; acute hepatitis; fulminant hepatic failure Use of herbal remedies and naturopathic substances should be investigated Onset < 1–12 months by drug administration	Normal or mild elevation	Variable	High	Moderate or severe	Loss of BSEP/MDR3 expression, canalicular cholestasis, hepatocellular inflammation
Low-Phospholipid Associated Cholelithiasis (LPAC)	ABCB4/MDR3	< 40y cholelitiasis; intrahepatic microlithiasis; recurrence of biliary symptoms after cholecystectomy; previous episodes of ICP; familial history of gallstones	High during obstruction	High	Normal or high	Normal or mild elevation	Not required; imaging-based diagnosis
Adult Cryptogenetic Cholestasis (ACC)	ATP8B1/FIC1 ABCB4/MDR3 ABCB11/BSEP TJP2/ZO-2	Itching for more than 6 months, lobular cholestasis	High	High	High	Normal	Lobular cholestasis (ductal hepatocyte metaplasia, ductal proliferation and immunohistochemistry for bile duct cytokeratin 7, anti-BSEP, and anti-MDR3 antibodies)
Hepatobiliary Cancer (HBC)	ABCB11/BSEP ABCB4/MDR3 TJP2/ZO-2	Hepatocellular carcinoma (HCC) Cholangiocarcinoma (CCA) Gallbladder and gallway cancer					

RIC, Recurrent intrahepatic cholestasis; ICP, Intrahepatic cholestasis of pregnancy; DIC, Drug-induced cholestasis; LPAC, Low-phospholipid associated cholelithiasis; ACC, Adult cryptogenetic cholestasis; HBC, Hepatobiliary cancer

### FIC-related genes and development of liver and biliary cancers

Primary liver cancer, the sixth most common cancer, usually occurs in conjunction with cirrhosis; 20% of these cases, nevertheless, may affect non-cirrhotic individuals.

HCC can occasionally be discovered during routine imaging tests without a known cause. Mutations in PFIC genes related to the transport and metabolism of BAs may be the cause of sporadic primary liver cancers in patients without identifiable liver disease or with cryptogenic cholestasis [9].

In paediatric and adult populations, FIC-related variants may result in HCC and CCA, especially for ABCB11 (about 5–15% of children with PFIC2 develop HCC in their second- third year of life) [9, 70], ABCB4 [15] and TJP2 [9]. Tumours with no other apparent cause can be related to FXR and MYO5B variants both in paediatric and non-paediatric populations [9].

Evidence collected via WES, WGS, and NGS [9] suggests a correlation between several genes implicated in the pathogenesis of PFIC, adult cholestasis (RIC, LPAC, ICP), and the risk of developing HCC and CCA in both children and adults: TJP2, FXR, MYO5B, SLC51B, SLC25A13, NOTCH2, JAG1, TGR5, ABCB11, ABCB4, and HNF1B [9].

A large-scale study [71] on the Icelandic population showed a strong correlation between some variants of ABCB4 and an increased risk of developing HBCs; the typical, non-pathogenic missense variant c.711A > T results associated with gallstones, ICP, cirrhosis, liver cancer, gallbladder cancer, and may represent a general risk factor for liver disease before 40 years. Since the discovered variants are not pathogenic, this emphasizes the role of benign variants in predisposing to HCC. Notably, the same variant ABCB4 c.711A > T was found to be significantly related to fibrosis progression and increased liver injury in patients with PBC [24].

Some PFIC gene variants in ABCB11, ABCB4, and TJP2 have been reported in patients with liver tumors; these data strongly suggest that subjects with idiopathic chronic cholestasis and personal or familial risk factors for inherited cholestasis, as well as DIC, ICP, or LPAC history, should be screened for a panel of primary cholestasis-related genes. These patients may also benefit from monitoring with periodic ultrasound exams. Interestingly, a tendency to present liver symptoms of PFIC-related genes in adults before the age of 40 can be observed [9, 42, 71] (Fig. 3).

## Diagnosis

The diagnosis of PFIC is based on a combination of clinical data and laboratory or biochemical results, radiologic and histological evaluations if needed [13], with the crucial support of genetic testing [2]. However, comprehensive guidelines on the genetic assessment of these pathologies are lacking, while on the other hand, the constantly evolving genetic tests may not be enough to provide a definitive result [72].

Biochemistry is the first step in the diagnostic process if the clinic suggests familial intrahepatic cholestasis; elevated BA levels, often associated with low γGT, can support the early diagnosis in children.

Genetic testing has become a secondary screening level for genetic causes in the neonatal cholestasis diagnostic algorithm [1, 7]. While the diagnosis of PFIC may start from clinical observation, diagnostic information is required to corroborate clinical suspicion. Genetic testing can aid in the differential diagnosis of PFIC [73], enable patients to benefit from innovative therapies [16], and provide appropriate genetic counselling to parents of affected children. A genetic test should be performed when suspicion of adult cholestasis or PFIC is raised.

According to the recently published EASL Clinical Practice Guidelines on genetic cholestatic liver diseases [68], genetic testing plays a crucial role in diagnosing cholestatic liver diseases. After ruling out more common causes of cholestasis in adults, genetic testing is recommended early in the diagnostic process for newborns and children with unexplained cholestasis. It should be considered in adults with unusual clinical features or those not responding to standard treatments. While genetic variants predominantly determine the phenotype of early-onset cholestasis, the association may be less evident in adult-onset cases [68].

Genetic testing is specifically recommended in both acute and chronic cholestasis presentations. In acute cholestasis, testing should be performed in cases of ICP, when serum bile acids are ≥ 40 μmol/L or if ICP occurs early (≤ 32 weeks of gestation), as well as in severe or recurrent DIC and unexplained recurrent episodes [74]. DIC is described in patients with variants resulting in BSEP and MDR3 deficiency. Patients should be offered genetic testing if they have a personal or family history of intrahepatic cholestasis, such as PFIC, BRIC, ICP, and LPAC combined with drug at-cholestasis risk use [20] (Fig. 2). In chronic cholestasis, genetic testing is advised after excluding primary biliary cholangitis (PBC) through auto-antibody testing and primary sclerosing cholangitis (PSC) via MRCP, as well as in atypical autoimmune liver diseases such as AMA/ANA-negative PBC or small-duct PSC. Additionally, screening for genetic cholestasis should be considered in patients with early-onset biliary lithiasis [75].

First-degree relatives of patients with MDR3 deficiency, given the higher risk of fibrosis/cirrhosis or liver cancer, should be screened genetically according to EASL recommendations [68, 71].

Additionally, the EASL suggests re-analysing the data at least every 3 years to identify newly discovered variants in patients who did not receive a diagnosis after the initial testing [68].

However, it is essential to remember that negative genetic test results do not necessarily rule out a diagnosis of PFIC or AOCD; therefore, the genetic test data must be consistent with the clinical phenotype, and the diagnosis must be considered in the presence of clinical symptoms. On the other hand, thorough research using WES revealed that variants that are not presently recognized as pathogenic are significantly frequent in adult patients with cholestatic symptoms, and carriers of these variants should be monitored for various liver pathologies, including tumours [71, 76, 77].

**Fig. 4:**
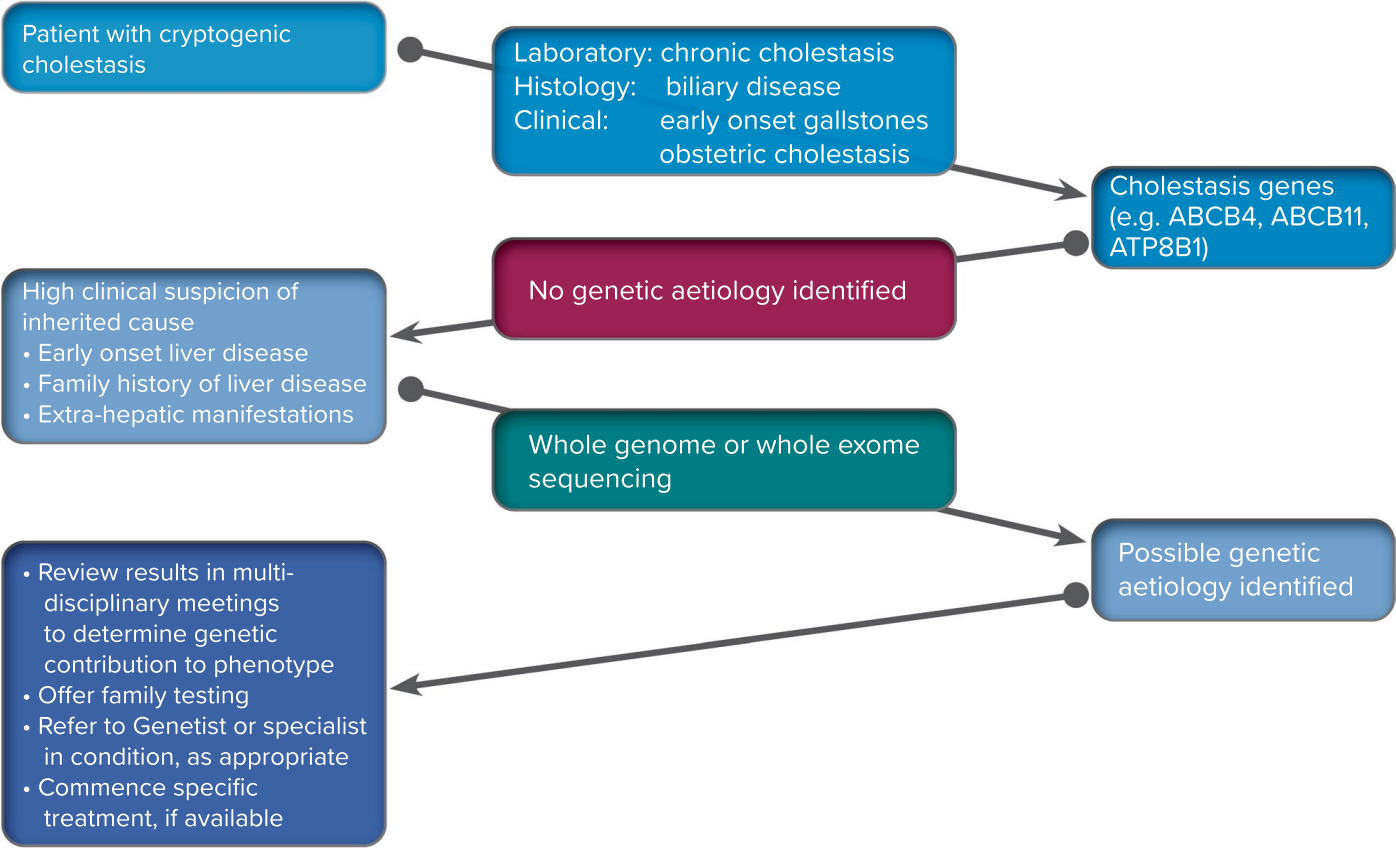
Proposed algorithm for genetic testing in patients with cryptogenic cholestasis

In a potential diagnostic flowchart (Fig. 4), a patient exhibiting clinical symptoms of cholestatic disease with no known cause should undergo genetic testing to look for mutations in the known genes that cause cholestasis [19].

Before making the diagnosis of PFIC, in the paediatric population, other cholestasis disorders should always be considered in the differential diagnosis, including biliary atresia, choledochal malformations, congenital endocrine disorders, inborn errors of metabolism (mainly during the neonatal period) as well as autoimmune liver diseases, drug-induced cholestasis and other genetic diseases in older children [72].

Biliary atresia represents the first cause of LT in childhood, and is the result of a rapidly progressive inflammatory and fibrotic process, with partial or complete obliteration of the extrahepatic and intrahepatic bile ducts; it is considered to be likely multifactorial [72].

Choledochal malformation (or congenital biliary dilatation) is a pancreaticobiliary anomaly characterized by dilatation of the biliary tract and, in most cases, a pancreaticobiliary maljunction. The diagnosis is based on the ultrasound, but it needs to be confirmed by cholangio-MRI [72].

Biliary atresia and choledochal malformations are to be surgically corrected immediately and should be excluded in a timely and appropriate manner in children [72].

Once biliary atresia, choledochal malformations, and infectious and secondary causes have been ruled out in a cholestatic newborn, the diagnosis is most likely associated with monogenic liver disease [72].

Elevated BAs in newborns and infants can be considered an extremely sensitive cholestasis biomarker. In adult patients, although their importance has long been recognized [78], the diagnostic role of BAs is unresolved [77]. One study found a correlation between BA levels and fibrosis in HBV [79], but overall, this examination seems underutilized. No studies have defined BA’s role in adult cholestasis aside from ICP [23].

Genetic testing for cholestasis-related mutations is recommended in paediatric and adult patients if the clinic, biochemistry, and histology suggest cholestasis. If the results do not allow for the precise identification of a genetic aetiology, and, still, the age of onset, extrahepatic manifestations, and family history of jaundice or ICP all point towards inherited cholestasis, a thorough investigation such as WES allows a high percentage of patients to reach a diagnosis.

WES is an effective tool for diagnosis in patients who remain undiagnosed despite a comprehensive clinical work-up [80]. WES remains vastly underutilized in non-oncological adult medicine, including in liver disease; a recent study showed that more than one-fourth of undiagnosed subjects had evidence of likely or definitive monogenic disorder as the cause of or significant contributor to their liver dysfunction using WES [80]; in another study, a third of a cohort of 52 patients with liver dysfunction of unknown aetiology was found to have genetic variations in liver disease-related genes [81]. Notably, this study’s patients under 40 were more likely to receive a genetic disease diagnosis. This higher diagnostic yield in younger patients is expected due to early-onset disease often associated with genetic variants that substantially impact the phenotype, making them easier to identify through genetic analysis [81]. Recently, exome sequencing, performed as a first-tier diagnostic test on a population of 299 children with intrahepatic cholestasis, allowed us to identify a new form of PFIC (PFIC 13) [37].

## Genotype/phenotype relation

As new genetic variants related to PFIC were discovered, genotype–phenotype relationships emerged [19]. In recessive diseases, the pathology is determined by variants (in homozygosity or heterozygosity) in both alleles; this is the universally accepted hereditary mechanism in classical PFIC. However, cases of cholestasis have been described in the presence of a single variant, suggesting a potential dominant transmission mechanism [19]. It is crucial to consider the patient’s genetic status (homozygous, heterozygous, or compound heterozygous) and the variants they carry when evaluating cholestatic phenotypes. Due to different mutations, patients with variants in the same gene may display a variety of phenotypes (“allelic heterogeneity”) [82]. After the introduction of NGS technology, many variants have been described for each PFIC-related gene, and new ones are frequently identified. To date, 554 variants are described in Clinvar [83] (a public database of reports on the relationships among human variations and phenotypes) for ATP8B1 [84]. According to the pathogenicity criteria from the American College of Medical Genetics and Genomics, the variants can be classified as pathogenic, likely pathogenic, benign, likely benign, or VUS [85], based on criteria using population, computational, functional data, and segregation data.

One of the main criteria for classifying variants is to categorize them into null variants, which result in a non-functional protein or no protein at all, and missense variants, which produce a protein with an altered amino acid sequence that may retain residual function, have reduced function, or even acquire a different function from the original protein.

Missense variants involve substituting a single amino acid, resulting in a protein with reduced or different functionality. Missense variants in FIC-related genes are associated with both PFIC and milder disease forms like BRIC, depending on the specific amino acid alteration and the patient’s genotypic status (homozygous, compound heterozygous, or heterozygous) [19].

Null variants (frameshift, nonsense, large deletions), which lead to a completely non-functional protein, are usually associated with PFIC [19].

To characterize a VUS, researchers need to verify its frequency by consulting the GnomAD database [86]. Variants with Minor allele frequency (MAF) over 1% are common variants (polymorphisms) non-disease-causing, while rare variants (MAF < 1%) need further evaluation [85]. However, a significant number of variants detected in patients with cholestatic liver disease fall into the VUS category, where it remains unclear whether the variant contributes to the patient’s phenotype [68]. Researchers should consult the primary databases [83], to evaluate any previous findings of variants, and to perform co-segregation studies. Creating clinical-genetic networks can help clarify the characteristics of VUS [87].

The different impacts of ABCB11 and ABCB4 variants illustrate the complex interactions between genotype and phenotype [19]. ABCB11 variants increase the risk of developing DIC and ICP, while individuals with ABCB4 variants are at risk for LPAC and ICP. Individuals with variants in ABCB11 rarely develop cholestasis until BSEP function drops below a threshold, which is also influenced by other factors (e.g., drugs, hormones); AOCDs, such as DIL or ICP, can develop when BSEP function falls below the approximate 25% threshold. On the other hand, individuals carrying ABCB4 variants that decrease MDR3 protein function have a more linear dose–response curve, reflecting the functionality of MDR3 [88].

ABCB4 mutations, involved in PFIC3, ICP, and LPAC syndrome, have mostly missense variants in heterozygosity in patients with late-onset diseases. These patients sometimes have liver cirrhosis but frequently have a milder clinical picture [89].

However, heterozygous adult ABCB4 carriers exhibit a wide range of clinical presentations. This variability persists despite their identical ABCB4 variant carrier status, implying that other factors—such as environmental influences—contribute to the observed phenotype [68].

For this reason, EASL guidelines suggest prudentially an individualized follow-up every three years for asymptomatic family members and first-degree relatives of heterozygous parents with severe MDR3 deficiency, even if they have normal laboratory values and no signs of advanced liver fibrosis or gallstone disease [68].

The study of the genotype/phenotype relation is complicated by the presence of numerous variants, often newly identified and not yet classified. Furthermore, the influence of hormonal factors linked to age, the presence of triggers, and the presence of hypomorphic variants (variants that produce a protein with reduced functionality) make this investigation complex. In a retrospective study, 365 patients who developed liver disease above 18 years of age, who underwent sequencing of cholestasis genes for therapeutic purposes were identified; 28.4% of patients had potentially disease-causing variants of ABCB4, ABCB11, and ATP8B1genes with different liver disease phenotypes. The study showed a correlation between genotype severity and phenotype gravity; regarding MDR3, the authors hypothesize a relatively linear relationship between protein function and progression of the clinical phenotype [22].

When routinely using NGS with targeted gene panels, one needs to be aware that although this method identifies numerous variations, the results may not be conclusive because the pathogenic gene might not be present in the commercially available panels. Commercially available NGS panels must contain genes known at the time of their marketing to be effective and must be updated as frequently as possible to include more recently identified genes. On the other hand, in cholestatic individuals in whom the NGS panels have not found pathogenic mutations, a comprehensive study like WES, performed over the full exome, can find potential gene candidates for a cholestasis association, as was the case with the most recently discovered PFIC, PFIC 13 [37, 90].

In such scenarios, additional testing, especially WES, is advisable to identify other mutated genes that might elude Targeted Resequencing.

## Treatment

The primary PFIC symptom, pruritus, is currently manageable with the proper treatment. This sometimes-devastating symptom for years has been controlled, albeit inconstantly and often not effectively, with drugs such as UDCA, cholestyramine, or rifampicin [13].

UDCA is the initial treatment for all PFIC subtypes. This hydrophilic bile acid is thought to counteract the potential hepatotoxicity of endogenous bile acids. It regulates the distribution of bile acids, lowers the cholesterol in the bile, and maintains the integrity of the mitochondria. It has cytoprotective, immunomodulatory, antioxidant, choleretic, and antiapoptotic properties [13].

Two-thirds of individuals with PFIC-3 and ABCB4 abnormalities respond well to UDCA; patients with mutations that lead to no MDR3 protein expression are not responsive to UDCA therapy [13].

SBD has been successfully used in patients with PFIC1 and PFIC2 who don’t respond to medical therapy and aren’t eligible for LT [13].

Concerning ABCB4 disease with at least one missense variant and a clinical phenotype with symptoms of hereditary cholestasis such as ICP, gallstone disease, LPAC ACC, DIC, UDCA therapy is recommended [91]. Patients carrying at least one missense variation, with a positive canalicular expression of MDR3 and a biliary phospholipid level over 6.9% of total biliary lipid levels, presented a better response to UDCA and more prolonged native liver survival [91].

IBATs approval for the treatment of PFIC has made the early diagnosis, supported by genetic screening, even more important both for paediatric and adult patients [16, 88] cases of patients successfully treated with IBATs, both in classic form and in rarer subtypes of PFIC [47, 53], even in the absence of a solid diagnosis of PFIC or in other cholestatic diseases, such as Alagille syndrome, have been described [92] supporting the primary role of clinical diagnosis. According to a recent position paper [88], unexplained cholestatic disease in children should raise suspicion of PFIC; genetic testing is recommended to confirm the genotype, laboratory tests, clinic evaluation, and QoL assessment. However, treatment with IBATs should be initiated as soon as possible [88] (even before genetic test results).

If the clinician chooses to use IBATs for these patients, their management should occur in an expert setting.

Additional investigation and guidance are needed for adult patients with idiopathic cholestasis [88]. There are few data on treatment efficacy, aside from occasional case reports [93] and case series [94] of adult patients treated with IBATs.

We need long-term follow-up data to confirm the promising roles of IBAT in changing the natural course of PFIC disease, such as the delay in the need for LT due to pruritus or end-stage liver disease. This aspect highlights the importance of global cooperation between pediatric and adult hepatologists [68].

However, in cases of severe acute intrahepatic cholestasis of adulthood triggered by drugs (including contraceptive agents), pregnancy, and intercurrent diseases, therapeutic plasma exchange has been explored as a treatment option. This approach has shown some potential benefits in managing severe cases and improving patient outcomes [68, 95, 96]. Considering the new knowledge, these patients could potentially be treated with IBAT inhibitors.

The availability of an approved drug could allow many paediatric patients to reach adulthood [97] without the need for LT and may require a new approach to addressing the transition from paediatric to adult hepatology. Transition is defined as an active, comprehensive, coordinated, individualized process focused on the needs of the adolescent suffering from a chronic pathology who is moving from paediatric to adult medicine. Transition requires adult hepatologists to thoroughly understand childhood cholestatic diseases and their relationships with adult cholestatic pathologies.

In animal models, gene therapy has produced encouraging results, particularly concerning ABCB4, whether through vectors expressing ABCB4 or by focusing on the disease mechanism [98].

## Conclusion

PFIC should now be considered part of a spectrum of diseases affecting paediatric patients, young adults, and older adults.

Adult hepatologists must increase awareness of these diseases because paediatric patients are increasingly likely to reach adulthood with their native liver, and because several adult cholestatic diseases are likely associated with the same genes as PFIC. Thanks to the availability of new, effective treatments, these disorders can be viewed as curable diseases, at least in terms of their most severe symptoms, always needing a specialized follow-up.

## Expert opinion

Research has transformed PFIC from diseases limited to the paediatric spectrum to disorders involving paediatric and adult patients. It is possible to move from the paediatric definition of PFIC to a notion of cholestatic disorders encompassing both adult and paediatric forms of cholestasis.

Recent therapeutic advances significantly impact the QoL of paediatric patients, who can reach adulthood by maintaining their native liver and avoiding surgery. Furthermore, the same genes causing PFIC determine many adult cholestatic diseases. For these reasons, adult hepatologists need to increase their knowledge of liver genetic diseases and the awareness that many of them are closely related and can share diagnostic and therapeutic approaches, and follow-up protocols.

Innovations in the treatment of PFIC, together with this paradigm shift, make the topic of the patient’s transition from paediatric to adult hepatology particularly relevant and generally highlight the need for collaboration between paediatric and adult hepatologists.

In-depth examination of the genetic connections among cholestatic diseases should be one of the main goals of short and medium-term research, to better understand the connections between the diseases’ underlying common causal mechanisms.

Diagnostic procedures can also be improved, with the introduction of shared protocols for assessment of BAs and the request for genetic testing, with particular attention to the reconstruction of the patient’s family history, and with a more precise role for instrumental diagnostics and histology. Specific biomarkers to assess disease prognosis and identify patients at higher risk of developing end-stage liver diseases and hepatobiliary complications after LT would be crucial. Sulfate BAs have been proposed and are currently being investigated as a potential biomarker for cholestatic disorders, especially for PFIC [99–102].

The main objective of future research is to increase the possibility of an early diagnosis. Research in this field can have the power to subvert the life expectancy of patients, ensuring them and their families a normal QoL. The evaluation of the long-term effect of the therapy with IBAT inhibitors on the progression towards fibrosis, cirrhosis of the liver, and oncological risk must be carefully investigated. Currently, the research aims to extend the therapy with IBATs to other pathologies, such as Alagille syndrome [103]; other important research should be performed to understand how to prevent the risks of neonatal mortality related to ICP and to avoid cholestatic episodes, such as RIC, becoming chronic. Some types of PFIC carry a significant oncological risk that needs to be further investigated; the long-term effects of new therapies on disease progression, including cancer risk, need to be investigated further [9].

PFIC has been introduced into the Neonatal Screening Programmes in Belgium (Baby Detect) by analysing dried blood spots collected in the earlier days of life [104, 105].

The introduction of PFIC into a neonatal screening program would bring benefits to potential patients, who would have access to drug treatment in a short time, but also to researchers who would have available epidemiological and clinical data otherwise difficult to obtain [106].

Cooperation between paediatric and adult hepatologists will be essential in the next years. Adult hepatologists will need to enter the world of PFIC to treat a more significant number of these patients. Surveys among hepatologists could help to understand the state of knowledge in this field and develop strategies for fostering cooperation. This could allow the implementation of personalized surveillance for early cancer detection and the assessment patients’ family histories, which contain important information on the inheritance of cholestatic diseases.

## Data Availability

Data sharing does not apply to this article as no datasets were generated or analysed during the current study.
